# Co-modulation analysis of gene regulation in breast cancer reveals complex interplay between *ESR1 *and *ERBB2 *genes

**DOI:** 10.1186/1471-2164-16-S7-S19

**Published:** 2015-06-11

**Authors:** Yu-Chiao Chiu, Chin-Ting Wu, Tzu-Hung Hsiao, Yi-Pin Lai, Chuhsing Kate Hsiao, Yidong Chen, Eric Y Chuang

**Affiliations:** 1 Graduate Institute of Biomedical Electronics and Bioinformatics, National Taiwan University, Taipei, Taiwan; 2 Greehey Children's Cancer Research Institute, University of Texas Health Science Center at San Antonio, San Antonio, Texas, USA; 3 Department of Medical Research, Taichung Veterans General Hospital, Taichung, Taiwan; 4 Bioinformatics and Biostatistics Core, Center of Genomic Medicine, National Taiwan University, Taipei, Taiwan; 5 Department of Public Health, National Taiwan University, Taipei, Taiwan; 6 Department of Epidemiology and Biostatistics, University of Texas Health Science Center at San Antonio, San Antonio, Texas, USA

## Abstract

**Background:**

Gene regulation is dynamic across cellular conditions and disease subtypes. From the aspect of regulation under modulation, regulation strength between a pair of genes can be modulated by (dependent on) expression abundance of another gene (modulator gene). Previous studies have demonstrated the involvement of genes modulated by single modulator genes in cancers, including breast cancer. However, analysis of multi-modulator co-modulation that can further delineate the landscape of complex gene regulation is, to our knowledge, unexplored previously. In the present study we aim to explore the joint effects of multiple modulator genes in modulating global gene regulation and dissect the biological functions in breast cancer.

**Results:**

To carry out the analysis, we proposed the **Co**variability-based **M**ultiple **Re**gression (CoMRe) method. The method is mainly built on a multiple regression model that takes expression levels of multiple modulators as inputs and regulation strength between genes as output. Pairs of genes were divided into groups based on their co-modulation patterns. Analyzing gene expression profiles from 286 breast cancer patients, CoMRe investigated ten candidate modulator genes that interacted and jointly determined global gene regulation. Among the candidate modulators, *ESR1, ERBB2*, and *ADAM12 *were found modulating the most numbers of gene pairs. The largest group of gene pairs was composed of ones that were modulated by merely *ESR1*. Functional annotation revealed that the group was significantly related to tumorigenesis and estrogen signaling in breast cancer. *ESR1*−*ERBB2 *co-modulation was the largest group modulated by more than one modulators. Similarly, the group was functionally associated with hormone stimulus, suggesting that functions of the two modulators are performed, at least partially, through modulation. The findings were validated in majorities of patients (> 99%) of two independent breast cancer datasets.

**Conclusions:**

We have showed CoMRe is a robust method to discover critical modulators in gene regulatory networks, and it is capable of achieving reproducible and biologically meaningful results. Our data reveal that gene regulatory networks modulated by single modulator or co-modulated by multiple modulators play important roles in breast cancer. Findings of this report illuminate complex and dynamic gene regulation under modulation and its involvement in breast cancer.

## Background

With the advances in DNA microarray and the Next-Generation Sequencing (NGS) technologies, transcriptomic profiling of biological samples can be obtained fast and cost effectively. The high-throughput genomic data enable systematic inference of gene regulatory networks (GRNs) [1,2]. In parallel, online databases, such as the Kyoto Encyclopedia of Genes and Genomes (KEGG) [3] and the Pathway Interaction Database (PID) [2], curate large volume of biologically (experimentally) validated gene regulatory pairs. These GRNs and pathways provide overall landscape of complex genome-wide gene regulation in biological systems. However, these gene regulatory relationships are typically derived under a single condition in a single cell line/tissue. From biological intuition, cells undergoing changes in cell cycle, environment, or cellular stress, and cells of different disease types or disease subtypes may recruit differential signaling pathways in response of cellular stimulation. Thus, strength and relationships of gene regulation are less likely to remain constitutive (unchanged) among these cells (reviewed in [4]). Ideker and Krogan proposed the scenario of "differential network biology" where GRNs and pathways can be massively rewired during adaptive cellular responses [5]. Notably, dynamic interaction among proteins was shown to be predictive of breast cancer outcome [6], implying that studying the dynamic changes in network topology, as the differentially expressed genes, can provide biological clues of complex diseases.

From the viewpoint of regulation under modulation, the dynamics of cellular conditions can be determined (modulated) by status of certain modulator genes. In other words, gene A regulates gene B under the modulation of C refers to the scenario where regulation strength between gene pair A and B is dependent on expression level of the modulator C. For instance, previous study identified genes that were predictive of patient prognosis of lung adenocarcinomas in the RAS signature dependent manner [7]. Also, competing endogenous RNA (ceRNA) regulation, referring to genes sharing common targeting miRNA that can regulate each other by competing for the limited pool of miRNAs [8-10], was shown to be modulated by expression levels of the common targeting miRNAs [11,12]. In breast cancer, Estrogen Receptor (ER) is the most well studied modulator in gene regulation. Topological and temporal changes in GRN of transcription factors were observed in MCF7 breast cancer cell line upon estradiol stimulation [13]. Furthermore, the ER encoding gene *ESR1 *was shown to be capable of modulating coexpression among a handful of genes [14]. In order to systematically investigate gene regulation modulated by individual modulator genes, comprehensive mathematical methods were developed and carried out biologically testable findings [9,15].

Gene regulation under modulation provides an alternative layer of gene regulatory networks. However, since gene regulation involves complex mechanism, especially in cancer, analysis based on individual modulator genes may be limited in understanding joint effects among multiple modulators and unveiling the landscape of modulation. Addressing this, in the present study we investigated the joint (cooperative, uncooperative, or dominant) effects of modulator genes in determining genome-wide gene regulation strength. Here we propose the **Co**variability-based **M**ultiple **Re**gression (CoMRe) method to model the relationships between multiple modulator genes and modulated gene-gene regulation in breast cancer. CoMRe was built mainly based on the multiple regression analysis which takes expression levels of modulators as model inputs and strength of gene-gene regulation, measured by our developed parameter "covariability", as output. On the other hand, investigation into functions governed by gene modulation in breast cancer remains largely unexplored. Thus, we further analyzed and interpreted the results identified by CoMRe in the systematic functional level. Collectively, the present study is aimed to statistically infer the relationship between multiple modulators and modulated gene regulation and to study the associated biological functions in breast cancer.

## Results and discussion

### Model overview of CoMRe

**Figure 1:**
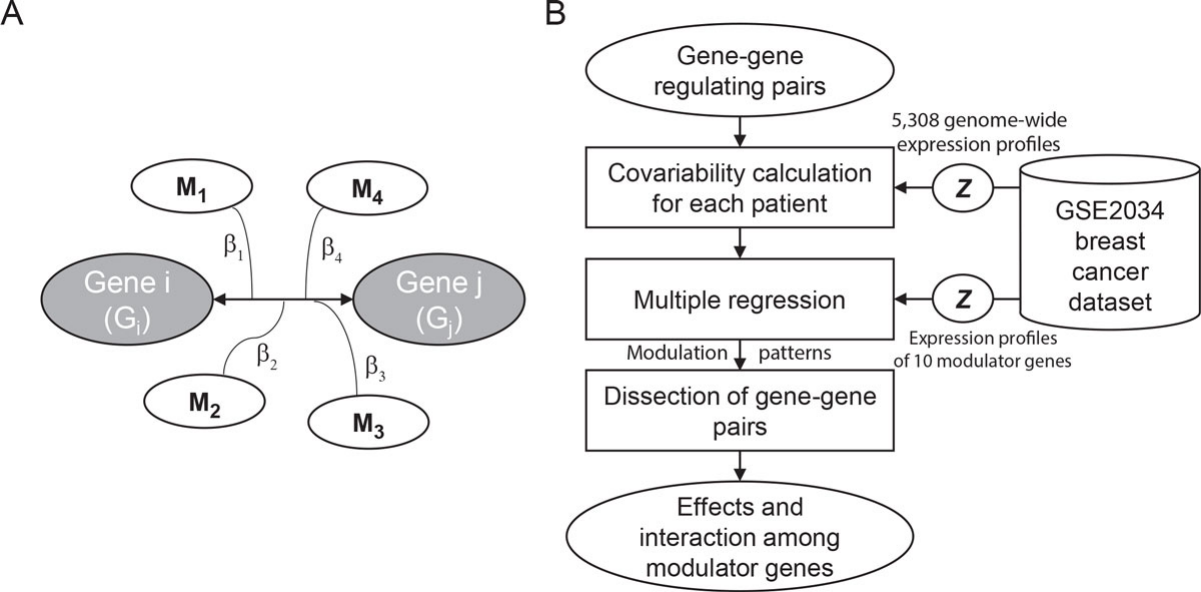
**Illustration of multi-modulator gene regulation and the CoMRe method**. (A) Illustration of multi-modulator gene regulation. From the point of view of regulation under modulation, regulation strength (left right arrow) between a pair of genes can be modulated by (***i.e*.**, dependent on) expression levels of some modulator genes. In the multi-modulator model, the modulator genes can have cooperative (or uncooperative) effects with differential capability (*β* values) in determining strength of gene-gene regulation. (B) Analysis flowchart of CoMRe. CoMRe is designed to infer the relationship between multiple modulator genes and modulated gene regulation from high-throughput datasets. Mathematically, CoMRe is composed of a multiple regression model that takes expression levels of the modulator genes as inputs and regulation strength between genes (measured by covariability) as output. Circled ***z ***in the figure stands for ***z***-transform. Mathematical details are described in the Methods section.

In the present study we aim to statistically infer the relationship between multiple modulators and modulated gene regulation (illustration in Figure 1A) and dissect biological functions governed by it in breast cancer. Here the modulated gene regulation refers to the scenario where regulation strength between two genes is specifically intensified when the modulator gene is highly up-regulated or down-regulated. We proposed the CoMRe algorithm to carry out the analysis. Figure 1B illustrates the analysis flowchart of CoMRe. The CoMRe algorithm is mainly composed of a multiple regression model that takes expression levels of the modulator genes as regressors (inputs) and the regulation strength of a modulated gene pair as regressand (output). Here we designed the "covariability" measure to model regulation strength between two genes in each sample. The covariability is simply the per-sample contribution into the Pearson correlation coefficient of genes *i *and *j*. From biological aspect, the covariability measures the magnitude of changes in two genes in the same direction in one sample; *i.e*., positive (or negative) covariability with greater magnitude is indicative of larger changes in two genes in the same (or opposite) direction. Mathematical details of CoMRe are provided in the Methods section.

In this study we analyzed the gene expression dataset from 286 lymph-node negative breast cancer patients (180 relapse free patients and 106 patients developing distant metastasis) (accession number GSE2034). After removing non-informative and background probe sets (detailed in the Methods section), we selected 5,308 probes representing 5,308 unique genes for consequent analysis. Current knowledge of modulator genes in breast cancer is very limited. Thus, besides the well-studied modulator gene estrogen receptor 1 (*ESR1*, the ERα encoding gene; as a positive result in this study), in the list of candidate modulator genes we exploratorily included 9 more candidate genes that were recorded as association with "breast tumor progression" in the knowledge-based database Ingenuity Pathway Analysis (Qiagen Inc.). Ten modulator genes analyzed in this study are listed in Table 1. These genes play crucial roles and execute complex functions in breast cancer; thus we reason their functions can be performed partially through modulation of gene regulation. We applied CoMRe to investigate effects of the 10 candidate modulator genes in modulating pairwise gene regulation in the microarray dataset. For each pair of genes, CoMRe outputs a co-modulation pattern, which is composed of ten-length vectors of regression *β* values and *p*-values. By studying all combinations of the 5,308 genes (14,084,778 gene pairs), we then elucidated the effects of individual modulator genes and the cooperative (or uncooperative) interaction among them in modulation. Furthermore, we explored enriched functions in the modulated gene pairs carrying distinct co-modulation patterns. To test the reproducibility of results identified by CoMRe, we also included two independent cohorts (accession numbers GSE4922 and GSE25066) as validation datasets.

**Table 1:** List of the 10 candidate modulator genes.

**Gene symbol**	**Entrez gene name**	**Location^a^**	**Type^a^**
*ADAM12*	ADAM metallopeptidase domain 12	Plasma Membrane	peptidase
*CCL5*	chemokine (C-C motif) ligand 5	Extracellular Space	cytokine
*ERBB2*	v-erb-b2 avian erythroblastic leukemia viral oncogene homolog 2	Plasma Membrane	kinase
*ESR1*	estrogen receptor 1	Nucleus	ligand-dependent nuclear receptor
*IGF1*	insulin-like growth factor 1 (somatomedin C)	Extracellular Space	growth factor
*MIF*	macrophage migration inhibitory factor (glycosylation-inhibiting factor)	Extracellular Space	cytokine
*MKI67*	marker of proliferation Ki-67	Nucleus	other
*MYC*	v-myc avian myelocytomatosis viral oncogene homolog	Nucleus	transcription regulator
*RECK*	reversion-inducing-cysteine-rich protein with kazal motifs	Plasma Membrane	other
*TP53*	tumor protein p53	Nucleus	transcription regulator

^a ^Gene information was obtained from Ingenuity Pathway Analysis (Qiagen Inc.).

### Dissecting individual effects of modulator genes in modulating gene regulation

**Figure 2:**
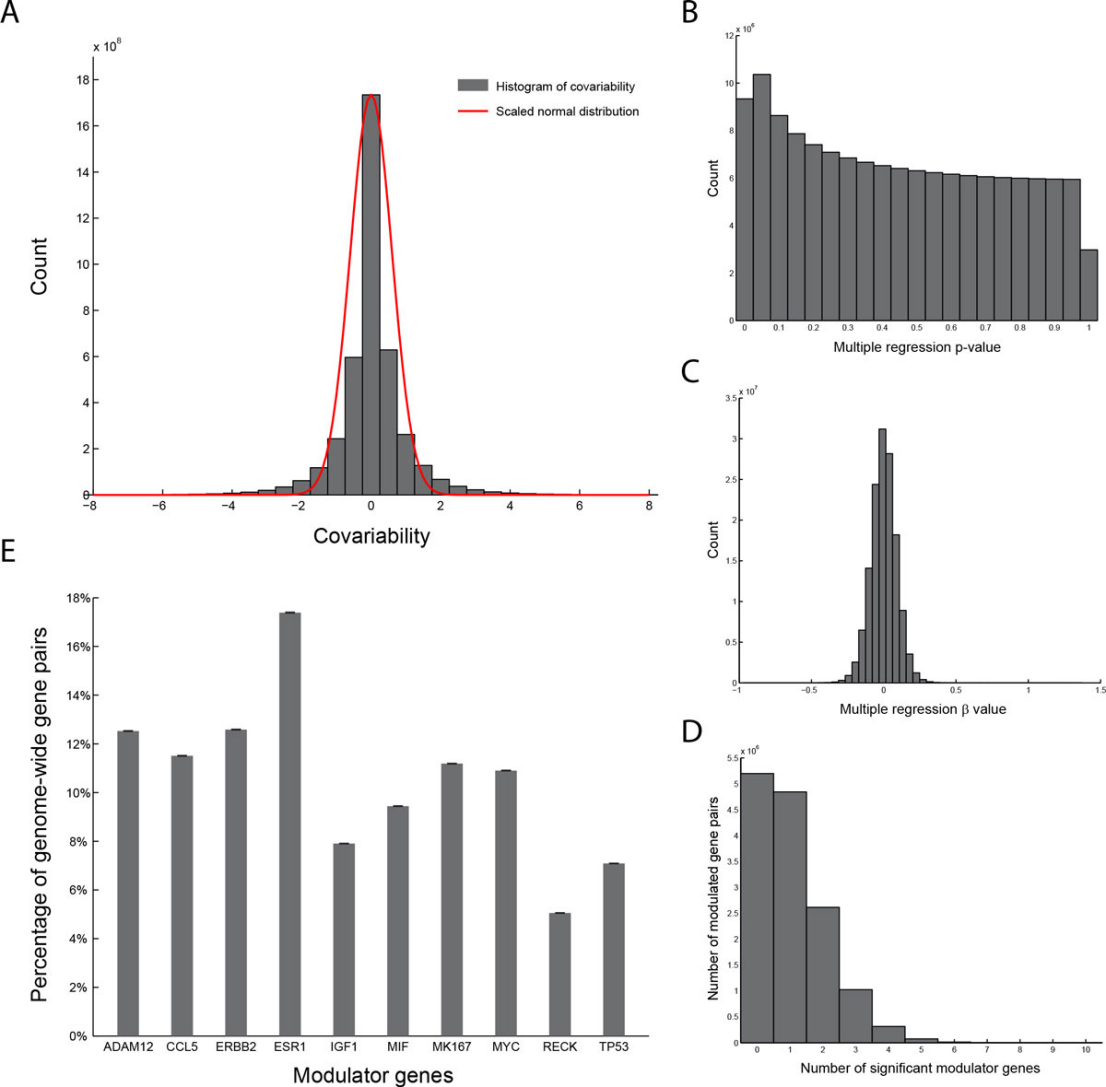
**Individual and cooperative effects of multiple modulator genes in modulating global gene regulation**. (A) Histogram of covariability. The covariability of 14,084,778 gene pairs in 286 samples (grey bars) were approximately normally distributed (red line), with the maximum, minimum, and mean values of 99.88, -77.83, and 0.03, respectively. (B) Histogram of multiple regression ***p***-values from CoMRe. The ***p***-values were significance levels of individual modulator genes in the multiple regression model and roughly followed the uniform distribution. (C) Histogram of multiple regression *β* values from CoMRe. The *β* values were approximately normally distributed. (D) Histogram of numbers of significant modulator genes among all gene pairs. 99.36% of all gene pairs were found modulated by less than 5 modulator genes. (E) Percentages of all gene pairs at which each modulator gene appears as a significant modulator. The percentage was calculated against 14,084,778, the total number of gene pairs. The well-studied modulator ***ESR1 ***was reported as the most significant modulator (in 17.39% of gene pairs), followed by ***ERBB2 ***and ***ADAM12***.

For each of the 14,084,778 gene pairs, CoMRe analyzes how the 10 candidate modulator genes interact to determine regulation strength between the pair of genes, and outputs a co-modulation pattern. The covariability of all gene pairs was approximately normally distributed, with the mean, maximum and minimum values of 0.03, 99.88, -77.83, respectively (Figure 2A). Among the 14,084,778 co-modulation patterns, regression *p*-values (significance of individual modulator genes in the multiple regression model) were roughly uniformly distributed (Figure 2B). The distribution of regression *β* values approximately followed the normal distribution (Figure 2C). Taken together, these observations indicate that the CoMRe method provides an unbiased statistical model. We set the criteria of multiple regression *p*-value < 0.05 to identify significant modulator genes for each gene pair. Regulation strength of 5,198,160 (36.91%) gene pairs was modulated by neither of the 10 candidate modulator genes. The other 8,886,618 (63.09%) gene pairs showed significant dependence on the total count of 14,871,721 candidate modulator genes; on average, each gene pair is modulated by ~1.67 modulators. A great majority (13,994,385 out of 14,084,778, 99.36%) of all gene pairs were modulated by less than 5 modulator genes. Figure 2D is the histogram of number of significant modulator genes in each gene pair. Interestingly, only one pair of genes, keratin 18 (*KRT18*) and N-acetylneuraminic acid synthase (*NANS*), was significantly modulated by all of the candidate modulators. Also, there were 9 pairs of genes modulated by nine of the ten modulators, including the pair of forkhead box A1 (*FOXA1*) and fructose-1,6-bisphosphatase 1 (*FBP1*). *FOXA1*, encoding a forkhead DNA-binding protein, is well-known to associate the luminal subtype and favorable prognosis in breast cancer [16-18]. *FBP1 *was reported to regulate epithelial-mesenchymal transition (EMT) in the basal-like subtype [19] and included in the widely used 70-gene expression predictor for breast cancer prognosis [20]. Altogether, we elucidate that *FOXA1 *may have highly modulated, thus "dynamic" across samples, regulatory relationship with *FBP1*, contributing to these two genes' roles in prognosis in different molecular subtypes of breast cancer.

Among the 10 candidate modulator genes, notably, the well-studied modulator gene *ESR1 *was found significantly modulating the most number of gene pairs (2,449,249 gene pairs, 17.39% of all pairs), followed by v-erb-b2 avian erythroblastic leukemia viral oncogene homolog 2 (*ERBB2*; 1,772,703 pairs, 12.59%) and ADAM metallopeptidase domain 12 (*ADAM12*; 1,764,441 pairs, 12.53%) (Figure 2E). Together with progesterone receptor (PR), ER and Her2 (*ERBB2 *encoded protein) are genes currently used for molecular subtyping of breast cancers. The results indicate that the two genes define distinct molecular characteristics in breast cancer partially through modulation of gene regulation. Among the modulator genes, reversion-inducing-cysteine-rich protein with kazal motifs (*RECK*), tumor protein p53 (*TP53*), and insulin-like growth factor 1 (*IGF1*), were found to modulate the least numbers of gene pairs (711,658 (5.05%), 998,142 (7.09%), and 1,113,258 (7.90%) gene pairs, respectively; Figure 2E). Although these genes are related to essential functions of breast tumor progression, they may possess relatively minor, or overtaken by other candidate modulators, effects in modulation of gene regulation. To generate a random baseline of our results, we replaced the inputs of modulator expression levels with ten randomly simulated variables and reran the analyses. Each of the ten random variables showed significance only in 3.33% to 5.81%, approximating the *p*-value cutoff of 0.05, of the 14,084,778 gene pairs. Taken together, our data suggest the capability of CoMRe in identifying both biologically well-known results and novel insights into other candidate modulator genes.

### Investigating joint effects of multiple modulator genes in modulating gene regulation and related biological functions

**Figure 3:**
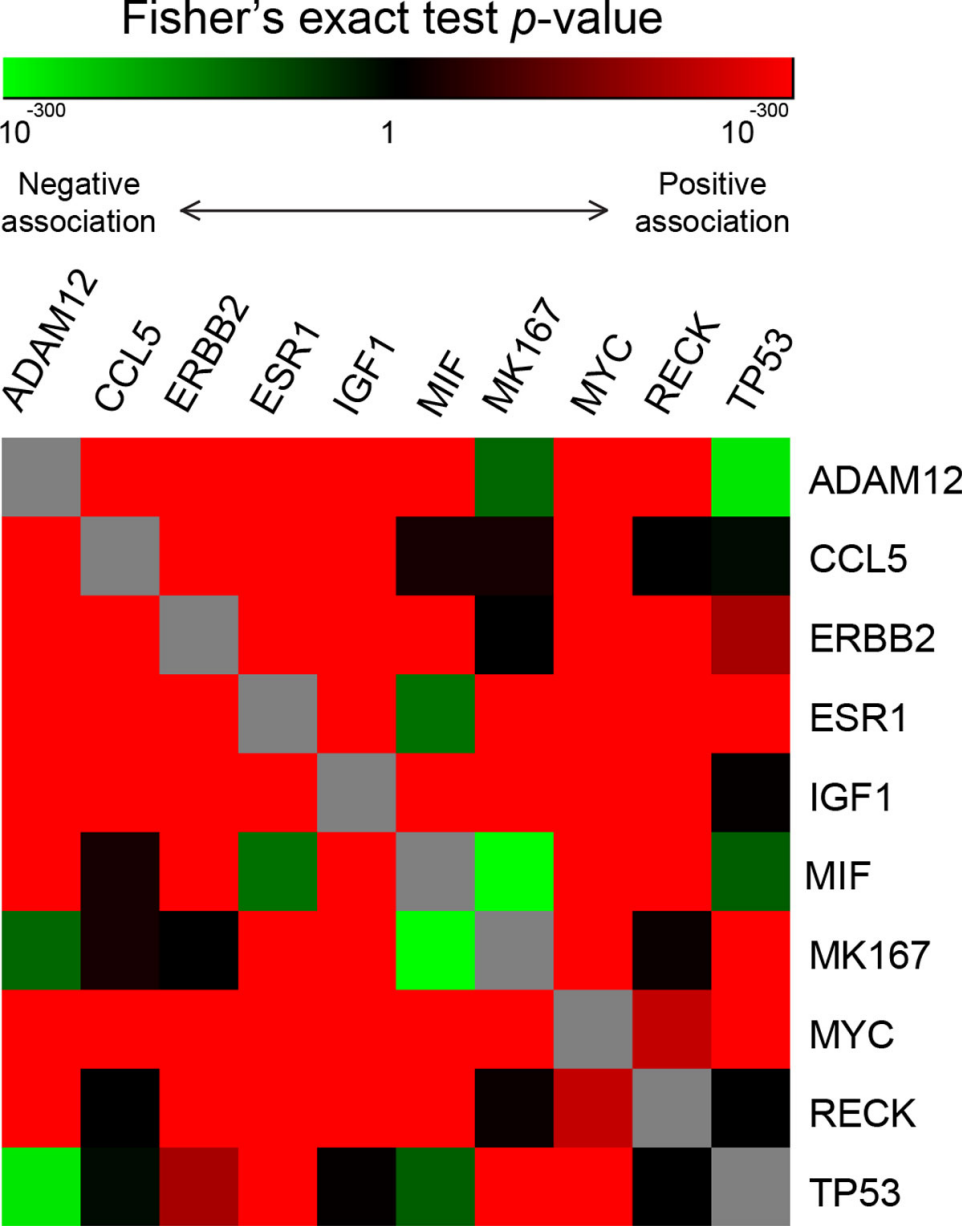
**Pairwise association between modulator genes**. Significance levels of pairwise co-occurrence of the modulators genes as significant modulators in all gene pairs are visualized using heatmap, with red and green denoting positive and negative association between two modulators, respectively. ***P***-values were obtained from Fisher's exact two-sided test and presented in the log-10 scale.

To understand the joint effects of the 10 candidate modulators, we analyzed pairwise co-occurrence as significant modulators among the genome-wide gene pairs; *i.e*., we statistically inferred whether gene pairs modulated by one modulator gene are highly overlapped with those modulated by another modulator. Interestingly, 36 (out of 45, 80.00%) pairs of modulators showed significant positive co-occurrence (Fisher's exact two-tailed *p*-value < 0.05); *i.e*., gene pairs modulated by one modulator tended to be also modulated by another modulator. Only 6 (13.33%) modulator pairs exhibited significant negative association (Figure 3), including *ADAM12*−*MK167, ADAM12*−*TP53, CCL5*−*TP53, ESR1*−*MIF, MIF*−*MK167*, and *MIF*−*TP53. CCL5*−*RECK *and *ERBB2*−*MK167 *showed negative pairwise association with borderline significance (both Fisher's exact *p*-values ~0.065).

We further grouped all the gene pairs based on their co-modulation patterns (significance of candidate modulators for each gene pair) so that gene pairs significantly modulated by the same set of modulators were grouped. Groups accounting for more than 5% of all gene pairs are tabulated in Table 2. In order to further realize underlying functions among genes in each group, we analyzed the parameter of node degree for genes as defined in graph theory. Node degree of a gene is defined as the number of first-order (direct) neighbor genes connected to it. Genes with high degrees are considered as "central" players (*i.e*., hub genes in Systems Biology) in a gene regulatory network. The top three genes with highest node degrees in each group are tabulated in Table 2. As we described above, the largest group (36.91% of gene pairs) was composed of gene pairs that were not modulated by any of the 10 modulators. Interestingly, groups of gene pairs that were modulated by single modulators (ranked from 2^nd ^to 11^th^) were found with higher frequencies than those modulated by multiple modulators. Gene pairs that were modulated merely by *ESR1 *were found as the second largest group (5.69%). Top 3 hub genes in the *ESR1 *modulated network were nuclear factor I/X (*NFIX*), sphingomyelin phosphodiesterase, acid-like 3A (*SMPDL3A*), and vascular endothelial growth factor A (*VEGFA*), with direct connection to 1,092, 1,072 and 1,045 nodes, respectively. In breast cancer, while function of *NFIX *was previously uncharacterized, *SMPDL3A *was reported to be dysregulated by progesterone treatment in hormone-independent breast cancer cells [21] and metastatic mouse mammary carcinoma cell lines [22]. Furthermore, *VEGFA *has been widely known for its roles in angiogenesis and endothelial cell growth. In breast cancer, studies demonstrated that *VEGFA *can prolong tumor cell survival [23] and its gene variation is associated with patient overall survival [24]. Our data further demonstrated that, potentially, the three hub genes' functions may be altered, fully or partially, under *ESR1 *modulation. To gain insights into biological functions governed by *ESR1 *modulation, we used the Database for Annotation, Visualization and Integrated Discovery (DAVID) v6.7 web tool to identify significantly enriched Gene Ontology (GO) terms of molecular functions and biological processes. For extracting biologically core information from the group, here only 10,235 "core" *ESR1 *modulated gene pairs (with Bonferroni adjusted *p*-value < 0.05 from CoMRe) composed of 964 genes were analyzed (Table 2). The top three clusters of enriched GO terms were i) DNA metabolic process and response to DNA damage, ii) identical protein binding, and iii) response to estrogen and steroid hormone stimulus (detailed in Table 3). During early tumorigenesis, effective DNA damage responses (the first cluster) can trigger cellular apoptosis and thus serve as candidate anti-cancer barrier [25,26]. Also, since response to estrogen stimulus (cluster 3) typically recruits downstream signaling genes of estrogen receptor, our results suggest *ESR1 *performs its functions, at least partly, by modulating these downstream genes. Overall, these data illuminated the importance of *ESR1 *modulation in breast cancer.

**Table 2:** Top co-modulation groups among the 10 candidate modulator genes.

**Co-modulation group**	**Number of modulated gene pairs**	**Percentage^a^**	**Top hub genes^b^**	**Number of core modulated gene pairs^c^**	**Number of core modulated genes^c^**
None of the 10 modulators	5,198,160	36.91%	*RALYL *(4253); *CALML3 *(4095); *KLK12 *(3920)	14,067,700	5,308
*ESR1*	801,229	5.69%	*NFIX *(1092); *SMPDL3A *(1072); *VEGFA *(1045)	10,235	964
*ADAM12*	579,710	4.12%	*LUM *(1005); *MYST4 *(993); *CHST1 *(988)	81	80
*CCL5*	556,253	3.95%	*CTSW *(1622); *CCL4 *(1209); *CCR5 *(1206)	3,168	405
*MK167*	537,860	3.82%	*CPS1 *(1160); *GDPD3 *(929); *KLHDC4 *(914)	251	241
*ERBB2*	519,905	3.69%	*MED1 *(1012); *PGAP3 *(1005); *STARD3 *(1000)	1,879	612
*MIF*	500,576	3.55%	*RPS2 *(1313); *FTL *(1200); *PRKCSH *(1137)	2	4
*MYC*	476,208	3.38%	*BOLA1 *(884); *FOSB *(844); *SLC38A2 *(817)	12	19
*TP53*	373,338	2.65%	*TP53 *(1636); *RAGE *(1005); *RABGAP1 *(909)	367	371
*IGF1*	266,990	1.90%	*PLA2G2A *(672); *TNXB *(662); *CIDEA *(659)	215	86
*RECK*	230,900	1.64%	*RECK *(1693); *DNAH3 *(1640); *OPHN1 *(1622)	459	137
*ESR1*−*ERBB2*	199,263	1.41%	*MIA *(780); *KRT6B *(631); *FYCO1 *(628)	407	141
*ESR1*−*MK167*	135,164	0.96%	*AZI1 *(753); *AQP5 *(688); *C3ORF37 *(515)	2	2
*ESR1*−*CCL5*	122,901	0.87%	*HOXA10 *(539); *PXDN *(465); *APOC2 *(462)	0	0
*ADAM12*−*CCL5*	109,845	0.78%	*CD38 *(661); *PRF1 *(660); *IL2RG *(544)	0	0
*ESR1*−*MYC*	104,650	0.74%	*KMO *(534); *ETFA *(394); *NAAA *(362)	0	0
*ADAM12*−*ESR1*	95,542	0.68%	*WNK1 *(290); *IGFBP4 *(288); *MFAP2 *(279)	0	0
*IGF1*−*MK167*	79,342	0.56%	*TTC23 *(436); *KIT *(306); *PRPF18 *(280)	0	0
*ADAM12*−*ERBB2*	76,759	0.54%	*MAP7 *(347); *VCAM1 *(270); *TRAPPC10 *(269)	0	0
*ADAM12*−*MYC*	72,520	0.51%	*FBN1 *(259); *MARCH5 *(241); *GADD45B *(234)	0	0
*ESR1*−*TP53*	71,837	0.51%	*CRLF1 *(565); *PGC *(435); *ITPKB *(317)	0	0

Only groups with frequency higher than 0.5% are listed.

^a ^Percentage of all 14,084,778 genome-wide gene pairs.

^b ^Top three hub genes (and the numbers of their first-order connected genes) in each modulated gene regulatory network.

^c ^Core modulated gene pairs/genes with Bonferroni adjusted *p*-value < 0.05.

**Table 3:** Top 3 clusters of enriched GO molecular functions and biological processes in *ESR1 *modulated genes.

**GO ID**	**GO term**	**Number of genes**	***P*-value^a^**
**Cluster 1 (Enrichment Score: 4.23)**			
GO:0006259	DNA metabolic process	54	7.85 × 10^-6^
GO:0033554	Cellular response to stress	55	8.80 × 10^-5^
GO:0006974	Response to DNA damage stimulus	40	1.32 × 10^-4^
GO:0006281	DNA repair	33	1.33 × 10^-4^
**Cluster 2 (Enrichment Score: 3.95) **			
GO:0042802	Identical protein binding	61	4.33 × 10^-5^
GO:0046983	Protein dimerization activity	52	1.48 × 10^-4^
GO:0042803	Protein homodimerization activity	36	2.21 × 10^-4^
**Cluster 3 (Enrichment Score: 3.66)**			
GO:0043627	Response to estrogen stimulus	19	1.98 × 10^-5^
GO:0010033	Response to organic substance	68	2.90 × 10^-5^
GO:0048545	Response to steroid hormone stimulus	26	7.27 × 10^-5^
GO:0009725	Response to hormone stimulus	38	3.94 × 10^-4^
GO:0009719	Response to endogenous stimulus	40	6.91 × 10^-4^
GO:0032355	Response to estradiol stimulus	9	0.010

Total number of *ESR1 *modulated genes (with Bonferroni adjusted *p*-value < 0.05): 964.

^a ^Modified Fisher's exact *p*-values from DAVID.

### Complex and tight interplay of *ESR1 *and *ERBB2 *modulation

**Figure 4:**
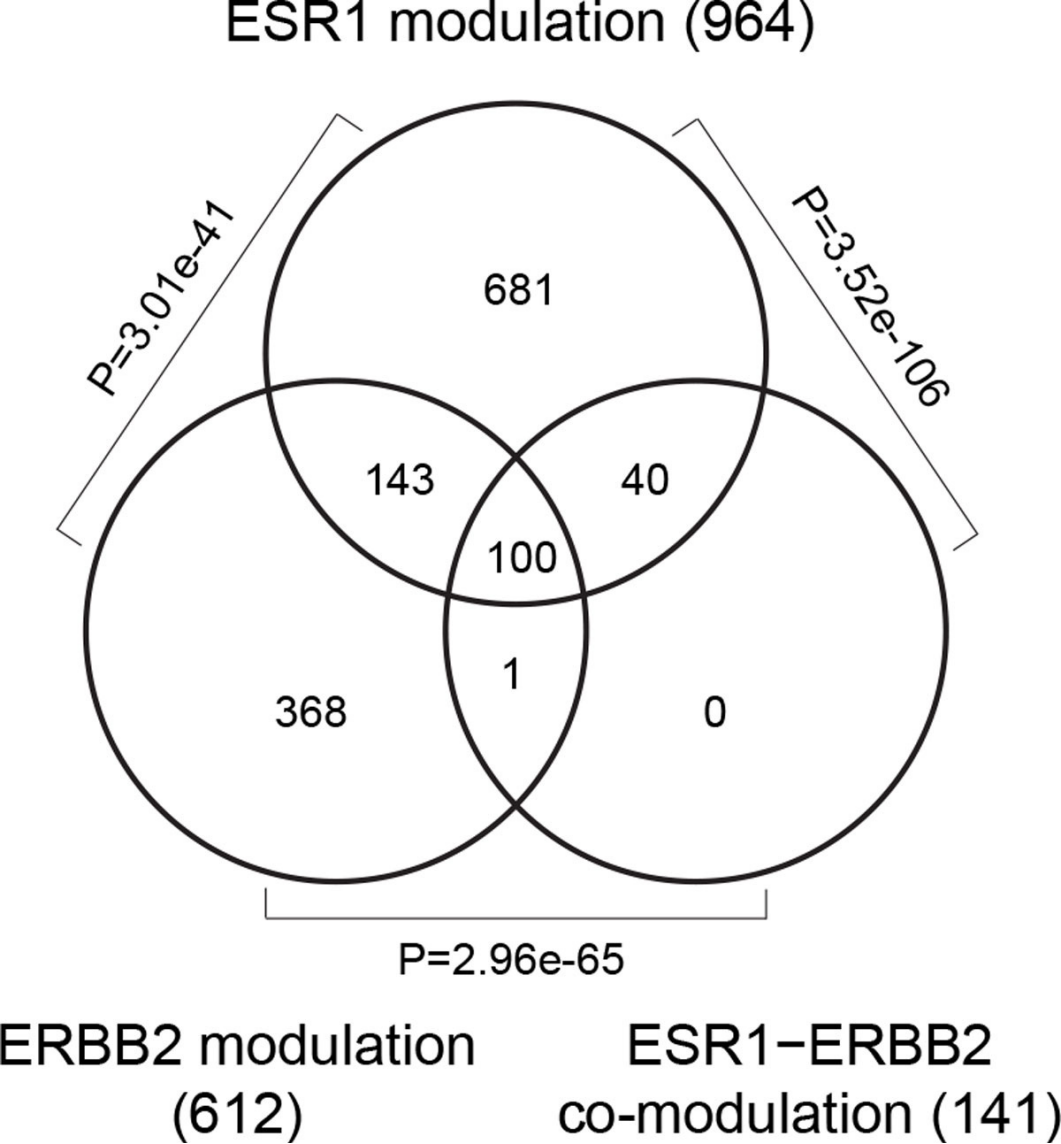
**Comparison of core *ESR1, ERBB2*, and *ESR1*−*ERBB2 *modulated genes**. The comparison is visualized by the Venn diagram of the core genes in the ***ESR1 ***alone, ***ERBB2 ***alone, and ***ESR1***−***ERBB2 ***co-modulation groups. All the genes in the co-modulation group were contained in at least one of the ***ESR1***- and ***ERBB2***-alone groups. Fisher's exact test showed these three groups shared highly similar gene contents.

In the list of groups of co-modulation patterns, *ESR1*−*ERBB2 *co-modulation was identified as the most frequent group with multiple modulators (Table 2). The group accounted for 199,263 gene pairs (1.41%) and had the top hub genes of melanoma inhibitory activity (*MIA*; 780 first-order neighbors), keratin 6B (*KRT6B*; 631 first-order neighbors); FYVE and coiled-coil domain containing 1 (*FYCO1*; 628 first-order neighbors). While the role of *MIA *remains unexplored in breast cancer, it is predictive of malignant melanoma progression and metastasis [27,28]. *KRT6B *is a basal specific marker in breast cancer [29]. Presence of and interaction between ER and Her2 define molecular subtypes of breast cancer and are associated with resistance to tamoxifen, a selective ER modifier (SERM) (reviewed in [30]). Here we showed that their encoding genes, *ESR1 *and *ERBB2*, also interact with each other and co-modulate regulation among a wide range of genes. Again, we used DAVID to analyze the 407 core gene pairs comprised of 141 genes in the *ESR1*−*ERBB2 *co-modulation group and identified similar results as in *ESR1 *modulation. The top three enriched groups of GO terms were i) response to hormone stimulus, ii) oxidation reduction and cofactor binding, and iii) identical protein binding (detailed in Table 4). Notably, the core modulated genes in the *ERBB2*-alone group were enriched in highly similar GO terms (data not shown). The core modulated genes of the *ESR1 *alone, *ERBB2 *alone, and *ESR1*−*ERBB2 *co-modulation groups were significantly overlapped (all pairwise Fisher's exact test *p*-values < 3.01 × 10^-41^, Figure 4). Interestingly, all of the *ESR1*−*ERBB2 *co-modulated core genes were included in the *ESR1 *or *ERBB2 *groups (Figure 4). Taken together, our data indicate that a highly common pool of genes is modulated by the two modulators while massive rewiring among these genes exists across different conditions (*i.e*., *ESR1 *or *ERBB2 *alone, and co-modulation). We elucidate that *ESR1 *and *ERBB2 *have complex and tight interplay in the aspect of gene modulation, through which identical biological functions are performed.

**Table 4:** Top 3 clusters of enriched GO molecular functions and biological processes in *ESR1*−*ERBB2 *co-modulated genes.

**GO ID**	**GO term**	**Number of genes**	***P*-value^a^**
**Cluster 1 (Enrichment Score: 2.22)**			
GO:0009725	Response to hormone stimulus	11	6.49 × -04
GO:0009719	Response to endogenous stimulus	11	0.001
GO:0048545	Response to steroid hormone stimulus	7	0.004
GO:0010033	Response to organic substance	14	0.004
GO:0043434	Response to peptide hormone stimulus	6	0.008
GO:0032868	Response to insulin stimulus	4	0.045
GO:0043627	Response to estrogen stimulus	4	0.051
**Cluster 2 (Enrichment Score: 1.92)**			
GO:0055114	Oxidation reduction	14	0.002
GO:0048037	Cofactor binding	7	0.012
GO:0050662	Coenzyme binding	6	0.012
GO:0009055	Electron carrier activity	5	0.089
**Cluster 3 (Enrichment Score: 1.83)**			
GO:0046983	Protein dimerization activity	11	0.008
GO:0042802	Identical protein binding	12	0.009
GO:0042803	Protein homodimerization activity	7	0.042

Total number of *ESR1*−*ERBB2 *co-modulated genes (with Bonferroni adjusted *p*-value < 0.05): 141.

^a ^Modified Fisher's exact *p*-values from DAVID.

### External validation of co-modulation patterns

In order to test the reproducibility and reliability of CoMRe among different cohorts, we analyzed two independent breast cancer datasets, GSE4922 and GSE25066, for validation. Based on the co-modulation patterns (*β* values of the modulator genes) obtained from GSE2034, we computed the "estimated" covariability profile for each patient in the two validation datasets using corresponding expression data of the modulator genes. The real covariability profiles were calculated using global gene expression data in each of the validation datasets. Notably, the estimated and real covariability profiles were significantly positively correlated (Pearson correlation *p*-value < 0.05) in 99.31% (287 out of 289, one-sample *z*-test *p*-value < computing precision of double-precision floating point, hereafter referred to as *p*-value ~0) and 99.80% (507 out of 508, *p*-value ~0) of patients in GSE4922 and GSE25066, respectively. Similarly, for *ESR1*−*ERBB2 *co-modulated gene pairs, the results were validated in 100% (all of 289 patients, *p*-value ~0) and 99.80% (507 out of 508, *p*-value ~0) of patients. The data suggest the stability of modulation effects among different cohorts and the reproducibility of results identified by CoMRe.

### Limitations and future work

By far validation of modulator genes through biological experiments is very limited. In breast cancer, ER is the most well-studied modulator gene. In the present study, in addition to the ER encoding gene *ESR1*, we exploratorily included 9 more genes related to essential functions in breast tumor progression, with previously undiscovered function of modulation. Our data first validated the role of *ESR1 *as a modulator gene and suggested that it may jointly work with other modulators. Also, the results implied the existence of other modulators genes in breast cancer, such as *ERBB2 *and *ADAM12*. However, ~37% of gene pairs were not modulated by any of the 10 candidate modulators, suggestive of the need for inclusion of other modulators. We have demonstrated the performance of CoMRe and the benefits to study modulation in the joint manner. With advances in biological exploration of modulator genes, CoMRe can be employed to reveal more biologically meaningful findings.

Investigation of casual relationships between genes is one of the crucial topics in regulatory biology. Indeed, correlation coefficients, as well as mutual information, are not capable of measuring causal relationships between factors. However, analyses of modulated gene regulation typically focus on how expression levels of modulators affect regulating strength, instead of the causal relationships, between modulated genes. Previous studies have used non-causal statistical methods to reach comprehensive results in single modulator analyses [9,15]. In this study, our objective is to extend the analysis to inferring multiple modulators co-modulated gene regulation, using a correlation-based regression approach. Therefore, CoMRe was designed to evaluate how co-variability of genome-wide gene pairs was dependent on modulator genes based on a multiple regression model; the analysis was focused on modulation, rather than direct or causal regulation, or co-regulation (*i.e*., regulated changes in gene expression levels).

CoMRe is built on the basis of a multiple linear regression model. In statistics, multiple regression analysis typically assumes the independence among input variables (*i.e*., expression profiles of modulator genes of CoMRe). However, biological intuition is that two genes can hardly be independent to each other in cells. In previous studies, multiple regression model has been widely utilized to study genes [31,32], different data types (from gene expression, transcription factor binding, and drug response data) [31,33,34], and survival significance of multiple genomic features (clinical subtypes and prognostic factors) [35,36]. Findings of these reports suggest that multiple regression can achieve biologically meaningful results, in spite of the moderate dependency of genomic features. Thus, we followed these literatures and designed CoMRe to study multi-modulator modulation. Future efforts may be spent on developing algorithms that can take dependent genomic features and enable statistically more meaningful inference.

## Conclusions

In the present study, we presented the CoMRe algorithm for systematically investigating how multiple modulator genes jointly determine pairwise regulation strength of modulated genes. The algorithm was designed based on a multiple regression model for gene-gene covariability that measures how two genes regulate each other in each patient. Among the ten candidate modulator genes, the positive control *ESR1 *and two genes with essential functions in breast cancer were found modulating the most numbers of gene pairs. Through functional annotation analysis, we showed that genes modulated by merely single modulator or co-modulated by multiple modulators play important roles in breast cancer. We elucidate that *ESR1 *and *ERBB2 *share complex interplay between each other in the aspect of gene modulation. We also demonstrated that the co-modulation patterns are stably retained and the results identified by CoMRe are highly reproducible among different cohorts. From the viewpoint of multi-modulator modulation, this study paves the way for better understanding complex gene regulation in breast cancer.

## Methods

### Microarray data

We analyzed gene expression profiles of 286 lymph-node negative breast cancers, of which 180 were relapse-free patients and 106 developed distant metastasis, from GSE2034 [37]. The samples were profiled with Affymetrix Human Genome U133A Arrays. We reprocessed the raw intensity values of CEL files using the Robust Microarray Analysis (RMA) algorithm into log-2 scaled probe set level expression levels. For multiple probe sets representing one unique gene, the one with the largest coefficient of variation (CV) was selected as the representative probe set. To eliminate computationally non-informative and background probe sets, probe sets with CV values < 5% or average expression levels < 6 (in the log-2 scale) across samples were filtered out from subsequent analysis.

We included two independent gene expression datasets for validation, composed of primary invasive breast tumors (NCBI/GEO Accession Number GSE4922 [38]) from Uppsala, Stockholm, and Singapore cohorts and pre-treatment invasive breast cancer patients in M. D. Anderson Cancer Center (GSE25066 [39,40]). 289 and 508 samples in the two datasets with complete molecular and clinical information were analyzed. The datasets were profiled with Affymetrix Human Genome U133A Arrays and we reprocessed the microarray data following identical procedures as described above. For each of the genes selected for analysis in GSE2034, one probe with the largest CV value in each validation dataset was extracted from the validation dataset for analysis.

### Covariability-based multiple regression

We generated a regression model that estimates the relationship between multiple modulator genes (assumed to be independent) and strengthen of regulation (*i.e*. correlation) between two modulated genes from the microarray dataset. To model the regulation strength of two genes (say, *i *and *j*) for patient *k *(totally *K *patients), we designed the "covariability" as

$$C_{i,j}^{k}=\frac{e_{i}^{k}-\mu_{e_{i}}}{\sigma_{e_{i}}}\cdot \frac{e_{j}^{k}-\mu_{e_{j}}}{\sigma_{e_{j}}}$$

where $e_{i}^{k}$ denotes the expression level of gene *i *in patient *k*, and $\mu_{e_{i}}$ and $\sigma_{e_{i}}$ represent the average and standard deviation of gene *i *across patients. Denotation of gene *j *is identical. The covariability was designed to measure the magnitude of changes in two genes in the same direction in one sample. Mathematically, it is simply the per-sample product-moment component in the calculation of Pearson correlation coefficient *ρ*; *i.e*., $\rho_{i,j}=\underset{k}{\sum }C_{i,j}^{k}$.

Based on the covariability, we proposed a multiple regression model to study the relationship between multiple modulator genes and covariability of gene pair. Given *M *modulator genes of interest, expression profiles of them are extracted from the microarray dataset and *z*-transformed (subtraction of sample mean followed by division of sample standard deviation) across samples so that each modulator approximately follows standard normal distribution. The *z*-transformation is employed to eliminate inter-gene systematic biases and allow the multiple regression model to give standardized coefficients for the modulators. Mathematically, the covariability-based multiple regression is modeled as

$$C_{i,j}=\underset{m\in M}{\sum }\beta_{m}e_{m}+\varepsilon$$

where the regressand $C_{i,j}={\left[C_{i,j}^{1}\;C_{i,j}^{2}\ldots C_{i,j}^{K}\right]}^{\prime }$ is the covariability vector of gene *i *and *j*, regressor $e_{m}={\left[e_{m}^{1}\;e_{m}^{2}\ldots e_{m}^{K}\right]}^{\prime }$ denotes the expression profile of modulator gene *m*, $\beta_{m}$ represents regression coefficients for modulator gene *m*, and  ε is the error vector. Statistical significance of the obtained regression coefficients was assessed using *t*-test. The regression model was iteratively applied to each combination of gene *i *and *j *in the microarray dataset. Thus, for each gene pair *i *and *j*, each modulator gene *m *takes a regression *β *value and *p*-value. A significant *p*-value indicates that the modulator is significantly predictive of the covariability (*i.e*., regulation strength) of corresponding gene pair. We defined the co-modulation patterns for each gene pair as the *M*-length vectors of *β *values and *p*-values for *M *modulator genes. To further dissecting the gene pairs based on their co-modulation patterns, we grouped gene pairs that were significantly modulated by the same set of modulators.

### Statistical analyses and functional annotation analysis

Fisher's exact test was employed to infer the significance of co-occurrence of significant modulator genes in the co-modulation patterns. Also, given a sample proportion $\hat{p}$, we estimated the 95% confidence interval of the population proportion by $\left[\hat{p}-1.96\sqrt{\frac{\hat{p}\left(1-\hat{p}\right)}{N}},\hat{p}+1.96\sqrt{\frac{\hat{p}\left(1-\hat{p}\right)}{N}}\right]$, where *N *denotes the sample size. To gain biological insights, we utilized the Database for Annotation, Visualization and Integrated Discovery (DAVID) v6.7 web tool [41,42] to identify the Gene Ontology (GO) [43,44] biological process and molecular function terms that exhibit significant enrichment in our gene list. In order to interpret the results in a more systematic and comprehensive level, we grouped highly overlapped GO terms into clusters using the DAVID Functional Annotation Clustering tool.

## Competing interests

The authors declare that they have no competing interests.

## Authors' contributions

YuC, CW, YiC, and EYC conceived the study together. YuC designed the analysis model. CW, YuC, and YL carried out the data analysis. YiC, TH, and CKH revised the study design. YuC and CW drafted the manuscript. YuC, YiC, and EYC revised and edited the manuscript. All authors read and approved the final manuscript.

## Declarations

The publication costs for this article were funded by the Greehey Children's Cancer Research Institute's intramural research fund.

This article has been published as part of *BMC Genomics *Volume 16 Supplement 7, 2015: Selected articles from The International Conference on Intelligent Biology and Medicine (ICIBM) 2014: Genomics. The full contents of the supplement are available online at http://www.biomedcentral.com/bmcgenomics/supplements/16/S7.
